# Supplementary material for: Potential of citizen science to advance urban planetary health research in low and middle-income countries: A scoping review
Source: PLOS Glob Public Health. 2025 May 7;5(5):e0003958. doi: 10.1371/journal.pgph.0003958 (PMC12057876; doi:10.1371/journal.pgph.0003958)
Supplement: S2 Table — (DOCX) [file pgph.0003958.s002.docx]

S2 Table. Search strategy and search strings

| **SEARCH STRATEGY** | | |
| --- | --- | --- |
| STEP ONE | | |
| Scopus and Web of Science databases: Aim – To find academic publications using the search strings in Annex  Scopus search on 03072024 = 115   1. All search strings below (title, abstracts, keywords) 2. Include last 10 years, English 3. Exclude: review, conference review, editorial, note, retracted, short survey, erratum 4. Limit to filter keyword: Citizen science   WoS search on 03.07.2024 = 170   1. All keywords in the Scopus search strings (topic - title, abstract, keywords) 2. Include last 10 years, articles, early access, proceeding paper, data paper 3. Exclude: review articles; editorial material; non-English 4. Limit to keyword within all fields - Citizen science   (Scopus) 115 + (WoS) 170 = 283 (Removed 53 duplicates) = 232 | | |
| Screening of titles  232 – 133 = 99 | Screening abstracts  99 - 77 = 38 | Full articles included = 22 |
| STEP TWO | | |
| Aim - To enhance the geographic distribution of articles after noting the limited distribution from WoS and Scopus above.  Search strategy:   - Use “Citizen science” with quotation marks to find an exact match AND underrepresented regions and countries. - Limit to last 10 years | | |
| Screening of titles = 20 | Screening of Abstracts = 15 | Full articles included = 9 |

| **SEARCH STRINGS** | |
| --- | --- |
| **Citizen science**  synonymes adapted from (2) | “Citizen scien*” OR “Citizen engagement” OR “non-professional scientist” OR “non-institutional participant” OR “non-academic participant” OR  “participatory research” OR “prosumers” OR “public participation” OR “community science” OR  “community-driven research” OR “community-based participation” OR  “volunteer*” OR “crowdsourc*” OR “open collaboration” OR "Citizen-based science" OR "citizen participation" OR "non-scientist" OR "non-researcher" OR “participatory map*” |
| AND | |
| **Planetary boundaries and Climate change**  Planetary boundaries synonyms adapted from: (3)  Climate change synonyms adapted from: (4) | "planetary health" OR "environmental health"  OR "ecosystem health" OR "biodivers*" OR "environmental change" OR "ecological health" OR "biodiversity" OR "environment*" OR "socio-ecological health" OR "natural system*" OR "anthropocene" OR "environmental determinant*" OR "planetary boundar*" OR "biosphere integrity" OR "land-system" OR "land-use" OR "deforest*" OR "land degrad*" OR "biogeochemical flows" OR "nitrogen cycle" OR "phosphorus cycle" OR "nutrient cycles" OR "ocean acidif*" OR "freshwater use" OR "water use" OR "water scarcity" OR "stratospheric ozone depletion" OR "ozone layer" OR "atmospheric aerosol loading" OR "aerosol pollut*" OR "air pollut*" OR “waste pollut*” OR "novel entities" OR "chemical pollution" OR "toxic chemicals" OR “climat*” OR "global warming" OR "greenhouse effect*" OR "greenhouse gas*" OR "carbon dioxide" OR “co2” OR “methane” OR “ch4” OR "nitrous oxide" OR “n2o” OR "nitric oxide" OR "nitrogen dioxide" OR “nox” OR “*chlorofluorocarbon*” OR “*cfc*” OR “refrigerant” OR “hydrofluorocarbon*” OR “hfc*” OR “*chlorocarbon*” OR "carbon tetrachloride" OR “ccl4” OR “halogen*” OR “ozone” OR “o3” OR “ammonia” OR “nh3” OR "carbon monoxide" OR “co” OR "volatile organic compounds" OR “nmvoc” OR "hydroxyl radical" OR "oh" OR "pm2.5" OR “aerosol” OR "black carbon" OR "organic carbon" OR "sulphur dioxide" OR "oxidi* sulphur" OR "so2" OR "sox" OR "sulphuric acid" OR “so4*”  OR “temperature*” OR “precipitat*” OR “rainfall” OR "heat ind*" OR "extreme heat event*" OR "heat-wave" OR "extreme-cold*" OR "cold ind*" OR “humidity” OR “drought*” OR “hydroclim*” OR “monsoon” OR "el nino" OR “enso” OR “SOI” OR "sea surface temperature*" OR “snowmelt*” OR “flood*” OR “storm*” OR “cyclone*” OR “hurricane*” OR “typhoon*” OR "sea-level" OR “wildfire*” OR "wildfire*" OR "forest-fire*" OR "coast*” OR "erosion" OR "coastal change*" OR “disaster*” |
| AND | |
| Urbanisation | "urban*" OR "city" OR "cities" OR "metropol*" OR "municipal*"  OR “subnational” OR “sub-national” |
| AND | |
| LMICs | "low-income country" OR "low-income countries" OR "middle-income country" OR "middle-income countries" OR "developing country" OR "developing countries" OR "emerging economy" OR "emerging economies" OR "low-resource setting" OR "low-resource settings" OR "LMIC" OR "LMICs" OR "Global South" OR "third world" OR "underdeveloped country" OR "underdeveloped countries" OR "less developed country" OR "less developed countries"OR "Afghanistan" OR "Albania" OR "Algeria" OR "Angola" OR "Argentina" OR "Armenia" OR "Azerbaijan" OR "Bangladesh" OR "Belarus" OR "Belize" OR "Benin" OR "Bhutan" OR "Bolivia" OR "Bosnia and Herzegovina" OR "Botswana" OR "Brazil" OR "Burkina Faso" OR "Burundi" OR "Cabo Verde" OR "Cambodia" OR "Cameroon" OR "Central African Republic" OR "Chad" OR "China" OR "Colombia" OR "Comoros" OR "Congo, Dem. Rep." OR "Congo, Rep." OR "Costa Rica" OR "Cote d'Ivoire" OR "Djibouti" OR "Dominica" OR "Dominican Republic" OR "Ecuador"  OR "Egypt, Arab Rep." OR "El Salvador" OR "Equatorial Guinea" OR "Eritrea" OR "Eswatini" OR "Ethiopia" OR "Fiji" OR "Gabon" OR "Gambia, The" OR "Georgia" OR "Ghana" OR "Grenada" OR "Guatemala" OR "Guinea" OR "Guinea-Bissau" OR "Guyana" OR "Haiti" OR "Honduras" OR "India" OR "Indonesia" OR "Iran, Islamic Rep." OR "Iraq" OR "Jamaica" OR "Jordan" OR "Kazakhstan" OR "Kenya" OR "Kiribati" OR "Korea, Dem. People's Rep." OR "Kosovo" OR "Kyrgyz Republic" OR "Lao PDR" OR "Lebanon" OR "Lesotho" OR "Liberia" OR "Libya" OR "Madagascar" OR "Malawi" OR "Malaysia" OR "Maldives" OR "Mali" OR "Marshall Islands" OR "Mauritania" OR "Mauritius" OR "Mexico" OR "Micronesia, Fed. Sts." OR "Moldova" OR "Mongolia" OR "Montenegro" OR "Morocco" OR "Mozambique" OR "Myanmar" OR "Namibia" OR "Nauru" OR "Nepal" OR "Nicaragua" OR "Niger" OR "Nigeria" OR "North Macedonia" OR "Pakistan" OR "Papua New Guinea" OR "Paraguay" OR "Peru" OR "Philippines" OR "Romania" OR "Rwanda" OR "Samoa" OR "Sao Tome and Principe" OR "Senegal" OR "Serbia" OR "Sierra Leone" OR "Solomon Islands" OR "Somalia" OR "South Africa" OR "South Sudan" OR "Sri Lanka" OR "St. Lucia" OR "St. Vincent and the Grenadines" OR "Sudan" OR "Suriname" OR "Syrian Arab Republic" OR "Tajikistan" OR "Tanzania" OR "Thailand" OR "Timor-Leste" OR "Togo" OR "Tonga" OR "Tunisia" OR "Turkmenistan" OR "Tuvalu" OR "Uganda" OR "Ukraine" OR "Uzbekistan" OR "Vanuatu" OR "Venezuela, RB" OR "Vietnam" OR "West Bank and Gaza" OR "Yemen, Rep." OR "Zambia" OR "Zimbabwe" |
