# Supplementary material for: Potential of citizen science to advance urban planetary health research in low and middle-income countries: A scoping review
Source: PLOS Glob Public Health. 2025 May 7;5(5):e0003958. doi: 10.1371/journal.pgph.0003958 (PMC12057876; doi:10.1371/journal.pgph.0003958)
Supplement: S3 Table — (DOCX) [file pgph.0003958.s003.docx]

| S3 Table. Data Extraction template | | |
| --- | --- | --- |
| Topic | Code | Discussion |
| **Background and Study characteristics** | |  |
| Research lead institution and country | Lead inst. & country | What is the correlation between lead institutions, sources of funding and the study context? |
| funding source or agency | funding |  |
| Study context – geographic location | geo context |  |
| **Methodologies** | |  |
| **CS Methods** |  |  |
| Demographics of Citizen scientists | Demo. CS | Age, sex, level of education, economic status |
| Demographics of study population | Demo. Study pop |  |
| Study design – CS recruitment | CS recruit | Existing partnerships, online platforms (social/legacy media), community outreach, word of mouth (also from a particular group of people eg high school students recruited from schools; runners recruited from running clubs etc) |
| Study design - CS training | CS train | Training approach (live demonstration of equipment use, instructions on how to conduct study) |
| Study design – CS compensation/remuneration | CS compens | Monetary, other incentives e.g certificates |
| Study design – CS motivation and retention | CS motiv | Community building, access to education and training, recognition and feedback |
| Engagement of CS in different levels of research from inception to conclusion of study | CS engage level | Manipulation, Therapy, Informing, Consultation, Placation, Partnership, Delegated Power, Citizen Control. Crowdsourcing, distributed intelligence, participatory science, extreme CS, contributory project, collaborative project, co-created project. Definitions of CS. |
| **Approaches to data** |  |  |
| Objectives and aims of the study | Study aim/obj | As stated by authors |
| Institutional approvals or permission to conduct study | Study permit |  |
| Data collection methods | Data collect method | Surveys, interviews, mapping, photovoice, specimen collection, sensing |
| Data collection tools/equipment and methods | Data collect tool | Paper-based, smartphone app, independent web platform, large aggregator platforms |
| Data analyses tools/equipment and methods | Data analyse tool |  |
| Data validation | Data valid | Training protocols, equipment calibration, expert verification, cross-validation, automated quality checks, comparison and replication. |
| Data storage | Data storage | Project-specific platforms, institutional repositories, cloud storage, aggregate platforms |
| Data access, privacy | Data access OR data priv | Paper-based, smartphone app, independent web platform, large aggregator platforms |
| Data dissemination and users | data sharing & feedback | Scientific publications, media coverage, project websites, dissemination workshops, open data portals, interactive maps/dashboards |
| **Planetary health dimensions** |  |  |
| Environmental health dimension | Environmental health | Using citizen science to measure environmental exposures related to any of these planetary health features: Biodiversity Air quality/pollution: capture pollutants measured (e.g. PM2.5, NOx, SOx, Ozone etc) and any discussion on sources Water quality/pollution and sources Waste pollution/management and sources Land use: green space Land use: open spaces Land use: recreational spaces Land use: walking/cycling infrastructure Land use: walking/cycling hazards Land use: hazards in the natural environment eg stagnant bodies of water Land use: food environment Green or blue infrastructure Water use/scarcity Food scarcity Chemical pollution including plastic Ecosystem Greenhouse gas emission: types and sources: industrial activity, construction, cooking etc Rainfall Flood Extreme weather: storm, fire (wild or forest), coastal erosion/change Other |
| Human health dimension | Human health | Mortality Morbidity Nutrition-related: Obesity/overweight, Underweight/stunting High blood pressure/hypertension Heart disease/stroke/CVD Injury/violence Heat-related illness ((e.g. heat stress, burn, exhaustion etc) Vector-born disease: e.g. malaria Water-borne diseases ( e.g. diarrhoea) Respiratory conditions (e.g. asthma, allergies etc) Mental health related (e.g. anxiety, depression etc) Health behaviour: physical activity (for leisure, travel, occupation) Health behaviour: diet-related Other |
| **Outcomes - Impact and Implications** | |  |
| Youth involvement | youth | In context of youthful LMIC cities |
| Opportunities for advocacy | advocacy | Engagement in decision-making and influencing policy |
| Inclusion considerations for equity and gender | Inclusion | From demographic data above (in addition to demographic alone, for gender, issues of gender equity e.g. considerations of how the measured exposures may disproportionately affect women. Or for age, discussions of intergenerational fairness) |
| Study limitations | Study limit | As stated by authors and in context of LMIC needs |
| Challenges and barriers faced during the implementation of citizen science, research gaps | barriers or challenges |  |
| Impact and recommendations for future studies | Study impact |  |
| Citizen science engagement |  | Categorisations and descriptions from 14 above. |
| Framing of planetary health |  | Frame 24 and 25 above using Lancet's work on planetary boundaries and climate change. |
