# Supplementary material for: Potential of citizen science to advance urban planetary health research in low and middle-income countries: A scoping review
Source: PLOS Glob Public Health. 2025 May 7;5(5):e0003958. doi: 10.1371/journal.pgph.0003958 (PMC12057876; doi:10.1371/journal.pgph.0003958)
Supplement: S4 Table — (DOCX) [file pgph.0003958.s004.docx]

S4 Table. Details of the data handling methods and tools from the reviewed studies

| **Author(s)** | **Data collection tools and methods by citizen scientists** | **Analyses methods by professional scientists** |
| --- | --- | --- |
| [7] | Soils samples collected using hand augers. Household surveys, assets and hazards mapping, structures and facilities mapping using satellite images on GPS tablets. | Lab analyses of soil samples. Thematic analyses of survey results |
| [8] | 15 low-cost optical particle sensors AlphaSense OPC-NC sensors (firmware version 18). Household survey. | Lab analyses of air quality measurements. Thematic analyses of survey results |
| [9] | Low-cost fixed and mobile air quality sensors submitted via the CanAirIO application. | Citizen scientists shared results directly to social media |
| [10] | Water quality was measured using colorimetric kit (www.alfakit.com.br). Benthic macroinvertebrates were collected by 2 community students using hand nets. | Analyses using indicator-screening process. An Integrated Disturbance Index (IDI) was calculated. |
| [11] | Cost-effective rain gauges fixed at households | Correlation analyses |
| [12] | Alabama Water Watch LaMotte Kit 21 to measure  physical-chemical parameters. | Lab analyses |
| [13] | FreshWater Watch kit developed by Earthwatch Institute to measure turbidity and nutrient variables. | Quantitative analyses |
| [14] | Manual collection, counting and classification of macro-litter items that were found within a sampling area. Recording on a datasheet. | Analyses done using R (version 4.3.0; R Core Team, 2023). |
| [15] | Collection of water samples. | Lab analyses |
| [16] | Horizontal coordinates were recorded using a handheld global positioning system. During the field survey, photos of the channel bed material, channel alignment, and land cover were captured. | Flow rate analyses |
| [17] | Graduated markers on a concrete surface or a combination of rope, stick and measuring tape to measure run-off. | Rating curves analyses |
| [18] | Photographs and descriptions sent to Google forms | Geographical distribution map using QGIS software version 3.20.1 |
| [19,20] | Photographs taken using mobile phones, digital cameras, or tablets and posted on Project website | Image analyses using the first operational version of the RIVeR (Rectification of Image Velocity Results) software. |
| [21] | BirdLasser mobile application and BirdLasser web platform (www.birdlasser.com) | Quantitative analyses |
| [22] | KoboToolBox (KoboToolBox.org) and KupuKita web platform (ww.kupukita.org) | Species analyses using Shannon-Wiener index and Simpson’s Index |
| [23] | Utrees mobile application and Website (www.utrees.cn) | Analyses using R (Version 3.6.2) |
| [24] | Stanford Neighbourhood Discovery Tool mobile application. 2. Actical accelerometers (version B-1, model 198-0200-03; Philips Respironics; Bend, Oregon). 3. Global Physical Activity Questionnaire (GPAQ, version 2) | Thematic analyses with citizen scientists |
| [25] | ArcGIS Survey123 mobile application with the Ecodatos form. | Records analyses using ArcMap |
| [26] | Epicollect5 smartphone application | Lab analyses |
| [27] | BirdLasser mobile application and BirdLasser web platform (www.birdlasser.com) | Visual distribution using ArcGIS |
| [28] | Case 1 - Data collected via smart phones using open-source software (Stakeholder WhatsApp platform). Case 2 - pictures of wildlife were uploaded onto iNaturalist platform (www.inaturalist.org) | case 1 - GPS coordinates of the incident locations enable the regulatory stakeholders to effectively identify the affected area for contingency response. Case 2 - Data was collated and analysed by the coordinating institutions. |
| [29] | Stanford Discovery tool application | Thematic analyses with citizen scientists |
| [30] | Pictures and audio narratives using Epicollect5 Mobile application. | Thematic analyses with citizen scientists |
| [31] | Photographs submitted through WhatsApp mobile application | Distribution maps using QGIS© 3.12.1 software. Predictions using Ecological niche modelling software MaxEnt |
| [32] | Interviews conducted through KoboToolBox application. | Thematic analyses with citizen scientists |
| [33] | Community walks and Conversation circles | Dialogic mapping with citizen scientists |
| [34] | Guided nature walks to collect data on howler monkeys and degraded native forests | Thematic analyses and prototyping with citizen scientists |
| [35] | surveys and interviews of focus groups with neighbourhood residents and key stakeholders. | Thematic analyses with citizen scientists |
| [36] | Manual forms filled with malaria relevant information and/or collecting mosquitoes using handmade carbon-dioxide baited traps. | Geographic data analyses using ArcGIS pro 2.4 |
| [37] | Semi-structured survey questionnaire was used to collect data | Thematic analyses with citizen scientists |
| [38] | Field sampling for biodiversity and ecosystem services potential of the green spaces, and an indirect perception survey | Descriptive statistics, statistical analyses, and visualisation of results using PRIMER version 7, Microsoft Excel or the R software |

Supplementary Figures References

1. Tricco AC, Lillie E, Zarin W, O'Brien KK, Colquhoun H, Levac D, et al. PRISMA extension for scoping reviews (PRISMA-ScR): checklist and explanation. Annals of internal medicine. 2018;169(7):467-73.

2. Haklay M. Citizen science and volunteered geographic information: Overview and typology of participation. Crowdsourcing geographic knowledge: Volunteered geographic information (VGI) in theory and practice. 2012:105-22.

3. Whitmee S, Haines A, Beyrer C, Boltz F, Capon AG, de Souza Dias BF, et al. Safeguarding human health in the Anthropocene epoch: report of The Rockefeller Foundation–Lancet Commission on planetary health. The lancet. 2015;386(10007):1973-2028.

4. Berrang-Ford L, Sietsma AJ, Callaghan M, Minx JC, Scheelbeek PF, Haddaway NR, et al. Systematic mapping of global research on climate and health: a machine learning review. The Lancet Planetary Health. 2021;5(8):e514-e25.

5. Arnstein SR. A ladder of citizen participation. Journal of the American planning association. 2019;85(1):24-34.

6. Vattakaven T, Barve V, Ramaswami G, Singh P, Jagannathan S, Dhandapani B. Best Practices for Data Management in Citizen Science-An Indian Outlook. Biodiversity Informatics. 2022;17:27-49.

7. Corburn J, Njoroge P, Weru J, Musya M. Urban climate justice, human health, and citizen science in Nairobi’s informal settlements. Urban Science. 2022;6(2):36.

8. Manshur T, Luiu C, Avis WR, Bukachi V, Gatari M, Mulligan J, et al. A citizen science approach for air quality monitoring in a Kenyan informal development. City and Environment Interactions. 2023;19:100105.

9. Barreneche C, Lombana-Bermudez A. Another Infrastructure Is Possible: Grassroots Citizen Sensing and Environmental Data Justice in Colombia. International Journal of Communication (19328036). 2023;17.

10. França JS, Solar R, Hughes RM, Callisto M. Student monitoring of the ecological quality of neotropical urban streams. Ambio. 2019;48:867-78.

11. Fehri R, Khlifi S, Vanclooster M. Testing a citizen science water monitoring approach in Tunisia. Environmental Science & Policy. 2020;104:67-72.

12. Calderón Cendejas J, Madrid Ramírez L, Ramírez Zierold J, Díaz Valenzuela J, Merino Ibarra M, Morató Sánchez de Tagle S, et al. Evaluation of the impacts of land use in water quality and the role of nature-based solutions: A citizen science-based study. Sustainability. 2021;13(19):10519.

13. Pérez-Belmont P, Alvarado J, Vázquez-Salvador N, Rodríguez E, Valiente E, Díaz J. Water quality monitoring in the Xochimilco peri-urban wetland: experiences engaging in citizen science. Freshwater Science. 2019;38(2):342-51.

14. De Veer D, Baeza-Álvarez J, Bolaños S, Araya SC, Darquea JJ, Poblete MAD, et al. Citizen scientists study beach litter along 12,000 km of the East Pacific coast: A baseline for the International Plastic Treaty. Marine Pollution Bulletin. 2023;196:115481.

15. Lekshmi B, Saha D, Sutar RS, Singh R, Prabhu SD, Kamat AM, et al. Science & Technology Agenda for Blue-Green Spaces Inspired by Citizen Science: Case for Rejuvenation of Powai Lake. Sustainability. 2021;13(18):10061.

16. Alemu AN, Haile AT, Carr AB, Trigg MA, Mengistie GK, Walsh CL. Filling data gaps using citizen science for flood modeling in urbanized catchment of akaki. Natural Hazards Research. 2023;3(3):395-407.

17. Mengistie GK, Wondimagegnehu KD, Walker DW, Haile AT. Value of quality controlled citizen science data for rainfall-runoff characterization in a rapidly urbanizing catchment. Journal of Hydrology. 2024;629:130639.

18. Pizá J, Spagnuoli JG, Dop NS, Cazzaniga NJ. From the Mediterranean to Yungas and Patagonia. Dispersal of the non-native gastropod Rumina decollata in Argentina. Biologia. 2023;78(3):865-71.

19. Le Coz J, Patalano A, Collins D, Guillén NF, García CM, Smart GM, et al. Crowdsourced data for flood hydrology: Feedback from recent citizen science projects in Argentina, France and New Zealand. Journal of Hydrology. 2016;541:766-77.

20. Le Coz J, Patalano A, Collins D, Guillén NF, García CM, Smart GM, et al., editors. Lessons learnt from recent citizen science initiatives to document floods in France, Argentina and New Zealand. E3S Web of Conferences; 2016: EDP Sciences.

21. Lee ATK, Nel H. BirdLasser: The influence of a mobile app on a citizen science project. African Zoology. 2020;55(2):155-60.

22. Winarni NL, Nuruliawati, Anugra BG, Junaid AR, Widayanti S, Aslan, et al. Can cities provide butterfly-friendly habitats? Biodiversitas Journal of Biological Diversity. 2023;24(4).

23. Yang J, Xing D, Luo X. Assessing the performance of a citizen science project for monitoring urban woody plant species diversity in China. Urban Forestry & Urban Greening. 2021;59:127001.

24. Odunitan-Wayas FA, Hamann N, Sinyanya NA, King AC, Banchoff A, Winter SJ, et al. A citizen science approach to determine perceived barriers and promoters of physical activity in a low-income South African community. Global Public Health. 2020;15(5):749-62.

25. Maillard O, Michme G, Azurduy H, Vides-Almonacid R. Citizen Science for Environmental Monitoring in the Eastern Region of Bolivia. Sustainability. 2024;16(6):2333.

26. Mitroi V, Ahi KC, Bulot P-Y, Tra F, Deroubaix J-F, Ahoutou MK, et al. Can participatory approaches strengthen the monitoring of cyanobacterial blooms in developing countries? Results from a pilot study conducted in the Lagoon Aghien (Ivory Coast). PLoS One. 2020;15(9):e0238832.

27. Tende T, Iniunam IA, Ivande ST, Awoyemi AG, Danmallam BA, Ringim AS, et al. Citizen science mitigates the lack of distributional data on Nigerian birds. Ecology and Evolution. 2024;14(4):e11280.

28. Zabbey N, Kpaniku N, Sam K, Nwipie GN, Okoro O, Zabbey F, et al. Could community science drive environmental management in Nigeria's degrading coastal Niger delta? Prospects and challenges. Environmental Development. 2021;37:100571.

29. Odeyemi E, Chesser S, King AC, Porter MM. Engaging Nigerian older persons in neighborhood environment assessment for physical activity participation: A citizen science project. Innovation in Aging. 2024;8(4):igad066.

30. Niyibizi JB, Nganabashaka JP, Ntawuyirushintege S, Tumusiime D, Umwali G, Rulisa S, et al. Using citizen science within an integrated knowledge translation (IKT) approach to explore cardiovascular disease risk perception in Rwanda. Frontiers in Tropical Diseases. 2021;2:752357.

31. Gervazoni P, Minuti G, Fuentes-Rodriguez D, Coetzee J, Sosa A, Sabater L, et al. Citizen science improves the known and potential distribution of a strong wetland invader: Implications for niche modeling and invasion management. Environmental Management. 2023;71(6):1176-87.

32. Croese S, Dominique M, Raimundo IM. Co-producing urban knowledge in Angola and Mozambique: towards meeting SDG 11. npj Urban Sustainability. 2021;1(1):8.

33. Pitidis V, Coaffee J, Lima-Silva F. Advancing equitable ‘resilience imaginaries’ in the Global South through dialogical participatory mapping: Experiences from informal communities in Brazil. Cities. 2024;150:105015.

34. Raño M, Palazzo MC, Soliz A, Holzer JC, Perez DA, Sánchez EM, et al. Community participatory action to build a canopy bridge for wild black and gold howler monkeys (Alouatta caraya) in northern Argentina. Folia Primatologica. 2022;93(3-6):453-63.

35. Mintchev N, Daher M, Jallad M, Pietrostefani E, Moore HL, Ghamrawi G, et al. Sustained citizen science from research to solutions: A new impact model for the social sciences. International Journal of Qualitative Methods. 2022;21:16094069221133232.

36. Murindahabi MM, Hoseni A, Corné Vreugdenhil L, van Vliet AJ, Umupfasoni J, Mutabazi A, et al. Citizen science for monitoring the spatial and temporal dynamics of malaria vectors in relation to environmental risk factors in Ruhuha, Rwanda. Malaria journal. 2021;20:1-18.

37. Okop KJ, Kedir K, Kasenda S, Niyibizi JB, Chipeta E, Getachew H, et al. Multi-country collaborative citizen science projects to co-design cardiovascular disease prevention strategies and advocacy: findings from Ethiopia, Malawi, Rwanda, and South Africa. BMC Public Health. 2023;23(1):2484.

38. Damptey FG, Opuni-Frimpong NY, Arimiyaw AW, Bentsi-Enchill F, Wiafe ED, Abeyie BB, et al. Citizen science approach for assessing the biodiversity and ecosystem service potential of urban green spaces in Ghana. Land. 2022;11(10):1774.
