# Supplementary material for: Potential of citizen science to advance urban planetary health research in low and middle-income countries: A scoping review
Source: PLOS Glob Public Health. 2025 May 7;5(5):e0003958. doi: 10.1371/journal.pgph.0003958 (PMC12057876; doi:10.1371/journal.pgph.0003958)
Supplement: Attachment — Submitted filename: Response to reviewer2 comments letter.pdf [file pgph.0003958.s008.pdf]

UNIVERSITY OF CAMBRIDGE

MRC Epidemiology Unit

*MRC Epidemiology Unit, University of Cambridge*
*Institute of Metabolic Science Building,*
*Cambridge Biomedical Campus,*
*Cambridge, CB2 0QQ*

*Tolu Oni*
*MBBS MRCP(UK) DFPH(UK) MPH(Epi) MD(Res) Mmed(Public Health) FCPHM(SA)*
*Clinical Professor*
*Director, Global Diet and Physical Activity Research Programme and Global Diet and Activity Research Network*

*Email: Tolullah.oni@mrc-epid.cam.ac.uk*

14 March 2025

Editor-in-Chief
Plos Global Public Health

Potential of citizen science to advance urban planetary health research in low and middle-income countries: A scoping review

Dear Editors

We thank the reviewers for the comments and provide detailed responses below.
I reconfirm that this manuscript has not been published and is not under consideration by any other journal. All authors have approved the manuscript and do not have any competing interests to declare.

With many thanks for your time and consideration.

Yours sincerely,

T Oni
On behalf of all authors

6. Review Comments to the Author

Please use the space provided to explain your answers to the questions above. You may also include additional comments for the author, including concerns about dual publication, research ethics, or publication ethics. (Please upload your review as an attachment if it exceeds 20,000 characters)

Reviewer #5: Thank you for this excellent and very interesting paper which is a great contribution to the field. I particularly enjoyed the breakdown of funding sources and institutional dominance, the discussion of how involved people were and also the different degrees to which the studies tried to achieve change as part of the study - this felt novel and very interesting to situate the studies within their own context and power dynamics.

General comments

**COMMENT**: The paper frames itself through the lens of the ‘Anthropocene’. Anthropocene as a term has been criticised for laying the blame for ecological destruction with generic references to ‘human’ activities, rather than explicitly naming an extractive economy. In reality, the timeframe referred to as the Anthropocene coincides with accelerated industrial/colonial capitalism, not the presence of humans, I would suggest using a different term or caveating the term Anthropocene with a reference to extractivism/the extractive economy (defined in the Movement Generation Just Transition book, for example) rather than human activities throughout the introduction and the paper in general. Similarly, Figure 2 refers to an escalation of ‘human pressure’ - again this falls into the same trap of placing equal blame on ‘humans in general’.

***RESPONSE**: We thank the reviewer for this response and valid perspective. The term “anthropocene” is a generally accepted term used in planetary health as “the proposed name for a new geological epoch demarcated as the time when human activities began to have a substantial global effect on the Earth's systems.” (Safeguarding human health in the Anthropocene epoch: report of The Rockefeller Foundation–Lancet Commission on planetary health. Whitmee, Sarah et al. The Lancet, Volume 386, Issue 10007, 1973 - 2028). This is the context within which it is used here. And we feel it is out of scope of this paper to deconstruct the merits or de-merits of this term (given that the focus of the paper is on citizen science methods for planetary health relevant research).*

**COMMENT**: Overall, the paper could benefit, in the introduction, analysis and conclusion, from increased discussion of the concept of accountability. Are the studies included seeking accountability? Are the issues being researched primarily imposed on the communities by global North corporations or other activities outside of the community? How is the burden of environmental harm disproportionate to resource use and contribution to the climate crisis? I think an increased consideration of accountability is extremely relevant given the focus on citizen science, where often communities are researching harm done to themselves and their environment for which they are not responsible, and given the references to planetary boundaries.

***RESPONSE**: We appreciate the reviewer’s interest in deepening the focus of this review. However this request is beyond the scope of this review; which is on the ways that citizen science methods are being used and reported in planetary health studies. We would welcome further research that builds on this review and explore the role of accountability. Indeed our rationale for recommending an approach to standardising reporting of citizen science research is informed by a paucity of data on different components of what conducting citizen science entails.*

**COMMENT**: The analysis, recommendations and conclusion frequently refer to professional researchers, and the importance of them training and validating the research produced by communities. What is not discussed in this paper is a process of mutual learning - how can citizen science be a way for professional researchers to learn from community expertise? How can it be a methodology which expands the possibility of including Indigenous knowledge systems into scientific research? Perhaps this was not a factor in any of the studies, and they remained top-down, but this is worth discussing more in my opinion, perhaps in the conclusion and recommendations if it was not part of the studies you found.

***RESPONSE**: We appreciate the reviewer’s interest in deepening the focus of this review. However this request is beyond the scope of this review; which is on the ways that citizen science methods are being used and reported in planetary health studies. We would welcome further research that builds on this review to explore the rationale for why citizen science is used and how that could complement indigenous knowledge systems as while out of scope for this review, these would be pertinent and welcome additions to the knowledge base on this topic.*

Specific comments

**COMMENT**: 24: ‘These settings are under going rapid urbanisation that would further threaten planetary boundaries’ feels out of sync with the proportion of responsibility for expanding planetary boundaries, I would suggest reframing this sentence regarding the specific health risks of rapid urbanisation and the ‘exposures mentioned’ e.g. pollution, overcrowding, overheating, etc, rather than centring planetary boundaries.

***RESPONSE**: The centering on planetary boundaries speaks directly to how we have defined planetary health-relevant research as this is the focus of the paper.*

**COMMENT**: 49: Exceeding planetary boundaries is not the only threat to human health. It is the process of exceeding those boundaries - i.e. the creation of pollution, deforestation and other ecologically destructive activities that harm human health. The introduction would benefit from mentioning the health relationships of the different planetary boundary indicators and the processes by which they are overshot, and clarifying the process of exceeding planetary boundaries also destroys health. Similarly, figure 2 focusses on the consequences, not the process of environmental degradation harming human health e.g. through oil spills and gas flaring which then leads to Co2 rise and the health harms of climate change. This is particularly relevant for citizen science which often measures the process e.g. of pollution, not only the outcome, of planetary change. A 'cradle to crave' analysis of extractivism may be helpful here - there is a Global Climate and Health Alliance report on this for the oil and gas industry which could be a helpful reference.

***RESPONSE****: We appreciate the reviewer's interest in deepening the planetary boundaries aspect of this manuscript. But as the focus of the paper is on the citizen science methods related to planetary health and not a review of planetary boundaries, further discussion on the points mentioned is beyond the scope of this work.*

**COMMENT**: 75: This paragraph cites the popularity of citizen science primarily as a result of its ability to collect large datasets and produce high quality research. I think it would be interesting to reference the history of citizen science as a necessity and a site of resistance and agency - where communities and activists have build their own research practices because public health has invalidated their experiences, especially Indigenous communities and communities affected by environmental racism.

***RESPONSE****: The scoping review typology requires a definition of the scope. We have elected to focus on the use of citizen science method in the majority world. An additional separate paper on the history of citizen science would be a notable addition to the knowledge base but beyond the scope of this project.*

**COMMENT**: 90: Similar comment re rapid urbanisation being implied as the main driver of environmental degradation in LMICS. Whilst environmental degradation is hugely and disproportionately accelerated in the global South, this paper would benefit from widening the scope of the the causes it sites for that reason, in particular referencing colonialism, neocolonialism, extractive industries, the electric/precious metals scramble, oil and gas expansion, and unequal climate impacts, for example.

***RESPONSE****: The focus of this review is on citizen science methods in planetary health research in order to strengthen the methodological rigor in conducting and reporting of such research. An additional paper on the history of citizen science and the causes of environmental degradation would be reasonable especially given the knowledge gap on this topic but beyond the scope of this review.*

**COMMENT**: 139: I would like to know more about the specific search terms / search strings / key words used in the study search, and more information as to what is classified as a 'planetary health relevant study' or 'citizen science projects that focus on promoting planetary health' an 'empirical study on citizen science relevant to planetary health in urban settings of LMICs' etc. Given that planetary health is a relatively new and field-specific term, there is a risk that this paper has excluded large areas of citizen science depending on which terms were used. In particular, I am thinking of the long history of the use of citizen science in the environmental justice movement. The paper would benefit from clarifying if environmental justice and associated search terms (such as environmental racism, Indigenous science, pollution) were incorporated with the keyword of citizen science, and reflection on if that affected the number of results.

***RESPONSE****: We kindly refer the reviewer to the supporting information document that extensively details the search terms and strings used.*

**COMMENT**: 483: Is it worth noting that none of the studies (as far as I can tell) measured health outcomes alongside environmental indicators such as water quality, biodiversity and air quality. The studies which measure human health outcomes do it in isolation, as do the studies which measure environmental indicators. I think it could be worth discussing and including in the conclusion - what would it look like to do citizen science that combined both exposure e.g. flooding, with peoples self reported health symptoms? How could tools like this be validated? What could this mean for seeking change?

***RESPONSE****: We thank the reviewer for this interest which is understandable given the dearth of synthesis on this topic. However it would unfortunately not be possible to address all aspects of a neglected topic to be covered in a single scoping review which is the first of its kind and focuses more on elucidating how citizen science methods are being used and reported in planetary health research. Our search terms were informed by environmental and health outcomes relevant to planetary health as defined in literature (see supporting information). To bound the scope of what could be a rather unwieldy paper, we have elected to focus recommendations on approaches to improve reporting of citizen science papers but welcome additional research from other on recommendations for tools to integrate exposures and outcomes; which would be valuable additions to the knowledge base*

**COMMENT**: 490: This discussion of political instability/political intervention would also benefit from a discussion re accountability - how does political instability intersect with the process of pollution and environmental extraction, therefore making the citizen science that could show that harm harder? Who benefits from the political instability making citizen science harder? Did any of the papers incorporate safety measures or other protection for citizen scientists? What challenge does citizen science pose to powerful interests and what are the implications for how to expand it?

***RESPONSE****: We appreciate the reviewer's interest in deepening the focus of this review. However this request is beyond the scope of this review; which is on the ways that citizen science methods are being used and reported in planetary health studies. We would*

*welcome further research that builds on this review and explore the role of accountability, power and instability in citizen science; a worthy addition to the knowledge base.*

**COMMENT**: 552: I would expand the environmental changes of planetary significance to be more specific of environmentally destructive activities such as deforestation, soil erosion, mining etc, rather than generic changes. I would add noise pollution to direct health effects and expand injury to include direct violence. You could potentially also add carcinogen exposure or cancer to direct health effects. I would expand aesthetics and culture to be more specific or give some examples, such as loss of traditional territory or access to sacred sites. I would add poverty and loss of livelihood. I would also include a reporting indicator on purpose of the study / each party's motivation in the study e.g. policy change. I would also include a reporting indicator on what safety, wellbeing check ups and support are available to citizen scientists.

***RESPONSE**: We thank the reviewer for this interest which is understandable given the dearth of synthesis on this topic. The selection of terms was informed by literature on the planetary boundaries (Whitmee S, Haines A, Beyrer C, Boltz F, Capon AG, de Souza Dias BF, et al. Safeguarding human health in the Anthropocene epoch: report of The Rockefeller Foundation–Lancet Commission on planetary health. The lancet. 2015;386(10007):1973-2028.) and research on climate and health (Berrang-Ford L, Sietsma AJ, Callaghan M, Minx JC, Scheelbeek PF, Haddaway NR, et al. Systematic mapping of global research on climate and health: a machine learning review. The Lancet Planetary Health. 2021;5(8):e514-e25.) as described in detail in the supporting information. Arguably there are several follow on papers that could follow including these reviewers' suggestions and we would welcome further research on the topics outlined by the reviewer. However these are beyond the boundaries of our review.*

**COMMENT**: 566: Majority World is a great term but only appears at the end of the paper - maybe could include at the beginning when you first introduce the term LMIC and suggest it as an alternative and then use consistently throughout the paper?

***RESPONSE**: We use this term at the end as LMIC definition was central to the search terms for this paper (see search terms) so felt it only appropriate to use the term in discussing findings rather than earlier.*

**COMMENT**: 600: I think the conclusion could be stronger regarding the potential of citizen science to fundamentally try to halt, change and repair environmental damage and planetary harm, not only to observe it. How can citizen science be a tool for empowering, supporting and engaging communities to in the challenges they face? How can it be a way to relate to a rapidly changing ecological system, and through that relationship repair the land and our own health?

***RESPONSE**: We thank the reviewer for this interest which is understandable given the dearth of synthesis on this topic. Arguably there are several follow on papers that could follow including these suggestions from the second set of reviewers: a review of the efficacy of citizen science, How citizen science can be a tool for empowering, supporting and engaging communities in the challenges they face? How it can be a way to relate to a rapidly changing ecological system, and through that relationship repair the land and our own health? etc. But these are out of scope of this review which at a first instance seeks to understand how citizen science is being used for planetary health relevant studies and to propose guidance on strengthening the methodology and standardise reporting to address some of the comparability and reporting deficits we encountered in our review.*

Reviewer #6: I appreciate the significant time and effort it took to gather this information. This manuscript has already gone through a round of reviews and edits/responses, which I will defer to those reviewers if they are satisfied with the authors' responses. I will make some other general comments:I do find this paper quite useful as a global health practitioner and environmental scientist. I particularly appreciate tables 5 and 6 with useful recommendations for standardization. I also appreciate the authors' defense of their methodologies. I realize that some of my comments may be outside of what can now be gleaned from the data, but I will still mention some areas I would be interested in more clarification if the authors/editors choose to explore them:

**COMMENT**: 1) what specific search terms were used? JUST citizen science for example, or were there synonyms for that term used. Same for "planetary health" or boundaries, etc. I know it says synonyms were used, but I am unclear on this and I would be curious to know those terms. If not, there may be studies absent from the review that termed their methods something different but with the same spirit of community involvement in data collection. I would be curious also to be able to conduct the search as a reader.

***RESPONSE**: We kindly refer the reviewer to the supporting information document that extensively details the search terms and strings used.*

**COMMENT**: 2) Did the included studies all conduct analysis on efficacy or were some only descriptions of project methodologies and how to engage the community in this work?

***RESPONSE**: We kindly refer the reviewer to the supporting information document that extensively details the inclusion criteria and data extraction template used.*

**COMMENT**: 3) I would be very curious to know more about the contexts for these studies.. WHY did they choose the citizen science methodology over other designs? Was it for resource reasons? For the sake of community engagement itself? For promotion of awareness in a community about an environmental issue? This may be too late to address in this manuscript, but as a global health/environmental health scientist working in LMICs, this would be a very useful addition for me.

***RESPONSE****: We thank the reviewer for this interest which is understandable given the dearth of synthesis on this topic. Arguably there are several follow on papers that could follow including these suggestions from the second set of reviewers: a review of the efficacy of citizen science, How citizen science can be a tool for empowering, supporting and engaging communities in the challenges they face? How it can be a way to relate to a rapidly changing ecological system, and through that relationship repair the land and our own health? etc. But these are out of scope of this review which at a first instance seeks to understand how citizen science is being used for planetary health relevant studies and to propose guidance on strengthening the methodology and standardise reporting to address some of the comparability and reporting deficits we encountered in our review.*

**COMMENT**: 4) What were the criteria for the studies being planetary health related? I see they were agreed upon by reviewers, but what were they. I see that some studies appear to only address a health topic (example: cvd risk), while others strictly environmental, while some had more obvious crossover. Were all studies related to health OR environmental topics included as long as they used citizen science methodologies - or were there very specific things that made them fall into the "Planetary Health" category?

***RESPONSE****: We identified studies that included health outcomes relevant to planetary health. As detailed in the methods, This study sits at the nexus of two main bodies of knowledge, - citizen science and planetary health and accordingly . We also kindly refer the reviewer to the supporting information document that extensively details the search terms used to answer the primary research question on methodologies used, and what human/planetary health outcomes are measured, in citizen science projects in urban LMIC settings?*

**COMMENT**: 5) I do agree with some of the previous reviewers that the discussion could be more in depth, but I realize the paper is already quite long and the authors are clear that their intent was more in aggregation of the information for future in-depth analysis. I would however love to see some discussion on the efficacy of this methodology. How much better or how much more data, or whatever the "more" positive outcome may be for the study by using citizen science methodology? What are the authors' impressions from the over 30 studies they analyzed? Is this a worthwhile investment of resources??

***RESPONSE****: We thank the reviewer for this interest which is understandable given the dearth of synthesis on this topic. However as identified by the reviewer, this is out of scope of a review that is already significantly lengthy.*
