# Supplementary material for: Potential of citizen science to advance urban planetary health research in low and middle-income countries: A scoping review
Source: PLOS Glob Public Health. 2025 May 7;5(5):e0003958. doi: 10.1371/journal.pgph.0003958 (PMC12057876; doi:10.1371/journal.pgph.0003958)
Supplement: Attachment — Submitted filename: Peer Review Report Dr. Awah Kum Tchouaffi.docx [file pgph.0003958.s005.docx]

**Peer Review Report: Potential of Citizen Science to Advance Urban Planetary Health Research in Low and Middle-Income Countries: A Scoping Review**

**1. Overall Impression:**

This scoping review makes a valuable contribution to the field by systematically exploring the use of citizen science in urban planetary health research within low and middle-income countries (LMICs). The paper effectively highlights the underutilized potential of citizen science in this context and identifies key methodological considerations and challenges. The call for standardized reporting and a focus on equitable funding is particularly timely and relevant. While the review is well-structured and written, some areas require further development to enhance its impact and rigor.

**2. Strengths:**

**• Clear Research Question and Scope:** The research question is clearly defined, and the inclusion/exclusion criteria are well-articulated, providing a focused and manageable scope for the scoping review.

**• Systematic Approach:** The authors followed established methodologies (PRISMA-ScR and JBI) for conducting a scoping review, enhancing the rigor and transparency of the process.

**• Relevant and Timely Topic:** The topic is highly relevant given the growing interest in planetary health and the potential of citizen science to address global health challenges, particularly in LMICs.

**• Critical Analysis of Funding:** The analysis of funding sources and the emphasis on the need for greater diversity and equitable funding distribution are crucial observations that contribute significantly to the paper's policy relevance.

**• Well-Structured and Written:** The paper is well-organized, easy to follow, and written, making it accessible to a broad audience.

**• Actionable Recommendations:** The authors provide concrete recommendations for future research and for standardizing reporting practices in citizen science planetary health projects.

**3. Areas for Improvement:**

**• Depth of Qualitative Analysis:** While the review identifies challenges and limitations, a deeper qualitative analysis of the included studies could provide richer insights into the contextual factors influencing the success or failure of citizen science initiatives. Consider exploring themes related to power dynamics, community engagement strategies, and the role of local knowledge systems.

**• Geographical Specificity:** While the review focuses on LMICs, further analysis of regional variations in the application and effectiveness of citizen science would strengthen the findings. Are there significant differences between regions or types of urban settings?

**• Discussion of Technology:** The role of technology in citizen science is mentioned, but a more in-depth discussion of the types of technologies used, their effectiveness, and their limitations (e.g., digital divide, data security) would be beneficial.

**• Integration of Grey Literature:** The review primarily focuses on peer-reviewed literature. Including relevant grey literature (e.g., government reports, policy documents, NGO reports) could provide a more comprehensive picture of the EIDM landscape.

**• Strengthening the Conclusion:** The conclusion could be strengthened by more explicitly linking the findings to specific policy recommendations and by outlining potential pathways for implementing these recommendations.

**4. Policy Implications:**

The findings of this scoping review have significant policy implications, particularly regarding funding allocation for citizen science initiatives in LMICs. The emphasis on the need for a greater diversity of funding sources and a shift in the center of gravity of funding decisions to better align with contextual realities in the Global South is a crucial policy message. The recommendations for standardized reporting will also improve the comparability and usability of citizen science data for policymaking.

**5. Overall Recommendations for Revision:**

**• Expand Qualitative Analysis:** Incorporate a more in-depth qualitative analysis of the included studies to provide richer insights into contextual factors.

**• Address Geographical Variations:** Analyse regional variations in the application and effectiveness of citizen science.

**• Deepen Technological Analysis:** Provide a more detailed discussion of the role of technology in citizen science initiatives.

**• Incorporate Grey Literature:** Include relevant grey literature to provide a more comprehensive overview.

**• Strengthen Conclusion and Policy Recommendations:** More explicitly link the findings to specific policy recommendations and outline potential pathways for implementation.

**• Consider Visualizations:** To improve readability and impact, enhance the presentation of data with clear and informative visualizations (e.g., maps, charts, and diagrams).

By addressing these points, the authors can significantly strengthen the manuscript and enhance its contribution to the field of citizen science and planetary health research. The paper has the potential to be a highly influential contribution to the literature.
