# Supplementary material for: Potential of citizen science to advance urban planetary health research in low and middle-income countries: A scoping review
Source: PLOS Glob Public Health. 2025 May 7;5(5):e0003958. doi: 10.1371/journal.pgph.0003958 (PMC12057876; doi:10.1371/journal.pgph.0003958)
Supplement: Attachment — Submitted filename: Response to reviewer comments.pdf [file pgph.0003958.s007.pdf]

MRC Epidemiology Unit, University of Cambridge
Institute of Metabolic Science Building,
Cambridge Biomedical Campus,
Cambridge, CB2 0QQ

Tolu Oni
MBBS MRCP(UK) DFPH(UK) MPH(Epi) MD(Res) Mmed(Public Health) FCPHM(SA)
Clinical Professor
Director, Global Diet and Physical Activity Research Programme and Global Diet and Activity Research Network

Email: Tolullah.oni@mrc-epid.cam.ac.uk

30 January 2025

Editor-in-Chief
Plos Global Public Health

Potential of citizen science to advance urban planetary health research in low and middle-income countries: A scoping review

Dear Editors

We thank the reviewers for the comments and provide detailed responses below.
I reconfirm that this manuscript has not been published and is not under consideration by any other journal. All authors have approved the manuscript and do not have any competing interests to declare.

With many thanks for your time and consideration

Yours sincerely,

T Oni
On behalf of all authors

REVIEWERS COMMENTS	AUTHORS' RESPONSES
**CROSS-CUTTING RESPONSE TO REVIEWERS**	We selected the scoping review method as this typology of literature review is appropriate and useful to map literature on evolving or emerging topics that have yet to be reviewed comprehensively and to identify gaps. The interest from the reviewers for more in-depth analysis underscores the importance and need for this scoping review as we agree that further work including more in-depth qualitative analysis, exploring of geographical variations and exploration of other contexts relevant to LMICs such as political instability. However, such additions would be beyond the scope of this initial scoping review. Instead, we have sought to respond to reviewers' comments with inclusion of ideas for further investigation in future studies and reviews as relevant.
**REVIEWER ONE**	
I suggest to carefully revisit findings and discussion section for easy understanding to readers.	We believe that by addressing the detailed feedback provided by the other three reviewers, we have significantly improved and more clearly articulated the findings and discussion sections.
**REVIEWER TWO**	
• **Depth of Qualitative Analysis:** While the review identifies challenges and limitations, a deeper qualitative analysis of the included studies could provide richer insights into the contextual factors influencing the success or failure of citizen science initiatives. Consider exploring themes related to power dynamics, community engagement strategies, and the role of local knowledge systems.	For this scoping review, such a qualitative analysis exploring power dynamics, community engagement, and role of knowledge systems is beyond the articulated scope that focused on overall trends and patterns across diverse citizen science initiatives.<br><br>However, the reviewer's comment regarding a deeper qualitative analysis to explore contextual factors is valid and suggests an important area for further research. We have added this in section *6. Conclusion and Recommendations.*
• **Geographical Specificity:** While the review focuses on LMICs, further analysis of regional variations in the application and effectiveness of citizen science would strengthen the findings. Are there significant differences between regions or types of urban settings?	We have explored this suggestion in the introduction section where we discuss the regional variations between the Global North and South. A more meaningful regional comparison is beyond the scope of our Scoping Review study hence we recommend further studies on regional variation in Section *6. Conclusion and Recommendations.*
• **Discussion of Technology:** The role of technology in citizen science is mentioned, but a more in-depth discussion of the types of technologies used, their effectiveness, and their limitations (e.g., digital divide, data security) would be beneficial.	The types of technologies used were well presented in the sub-section *3.7. Data tools and Methods.*<br><br>We have added more insights on the effectiveness and limitations of these technologies with reference to the digital divide and data security under the sub-section *The potential of Technology* in the Discussion section.
• **Integration of Grey Literature:** The review primarily focuses on peer-reviewed literature. Including relevant grey literature (e.g., government reports, policy documents,	We had included grey literature in the *Introduction* section.<br>We also explained in the *Materials and Methods* section that the grey literature was not included in the scoping review as they did not present substantial empirical material for the review.

NGO reports) could provide a more comprehensive picture of the EIDM landscape.<br><br><br><br>**• Strengthening the Conclusion:** The conclusion could be strengthened by more explicitly linking the findings to specific policy recommendations and by outlining potential pathways for implementing these recommendations.	This notwithstanding, we appreciate this suggestion and have added more grey literature to enhance the policy recommendations and strengthen the conclusion as below:<br><br>In the sub-section *Funding and Lead Institutions* we have added a paragraph on how policy and regulatory instruments can further enhance leadership of Global South institutions in citizen science projects, using the example of Argentina.<br><br>In the conclusion, we have also added policy recommendations on enhancing citizen science with examples from Argentina, India, and the African Union.
**• Consider Visualizations:** To improve readability and impact, enhance the presentation of data with clear and informative visualizations (e.g., maps, charts, and diagrams).	While we agree that in general visualizations like maps, charts, and diagrams can enhance data presentation, the methodologies and data in the reviewed citizen science projects vary too widely that the results do not lend themselves well to traditional visualizations. As such, we present the results in tables to ensure readability. For instance, regarding geographic distribution, we considered mapping the study locations against lead institution locations, and funding sources. This map did not yield a useful map as the three data points were highly varied in each case. In data collection,some projects gathered highly qualitative data such as narratives and community perspectives, while others gathered numerical data such as air quality or water quality measurements. Similarly, the data tools varied from paper-based forms to highly specialised proprietary equipment. These variations do not lend well to generalisations on charts or diagrams.
**REVIEWER THREE**	
Although you selected significant features of citizen science in the articles, but did not discussed clearly how much participatory those studies were. Citizen science is ideal to reverse the gaze towards community's perspectives but the projects using citizen sciences often adopt same top-down approach for selecting the design and participants. You may like to reflect more on this bias in your analysis.<br>I would also like to see your reflection on challenges of these projects faced for engaging citizens and how they overcome participation bias.	We agree that participation is a significant feature of citizen science. Hence, we discussed at length the sub-section *3.5 levels of engagement* as a sub-section in the methodologies section. To further highlight the participatory aspects of the reviewed studies, we have renamed this sub-section as *Participation and engagement of citizen scientists.*<br><br>A significant finding was that many papers did not adequately report on the extent of participation and their approaches to overcoming participation bias. This data paucity and lack of standardised approach to reporting participation informed our recommendations for a standardised approach to reporting “Level of Engagement” and “Challenges and Limitations" in Table 5.
**REVIEWER FOUR**	

Line 51: "Recent assessments…" Can you provide a citation for this? If it is (4), I would also suggest indicating that there.	The reference is (4) and has been inserted.
Line 59-63: Should the (6) definition be block text?	The definition from the Lancet Commission on Planetary health is in quotation marks hence no need to be in block.
Line 66: I believe the authors mean 'Figure 1' here? Figure 2 appears later in the manuscript, while Figure 1 appears first.	We did intend this to be figure 2 which appears at the end of the paragraph hence the reference was correct and remains as such.
Line 146: This is where the other figure error is. Please revise. The diagram is clear in steps. It is a bit blurry, but I assume that is how the submission usually appears on a manuscript submission.	The reference correctly refers to figure 3 which is the "Search and screening strategy."
Line 67: Would examples of the escalation of human pressure in parentheses be of help here? Also, when I look at the figure, I am left wanting details of what those are. Examples of them somewhere, either the body, or in the figure, would help.	We have added examples of human pressures such as climate change, ozone depletion, biodiversity loss, and urbanization, which are listed in full in figure 2.
Lines 22 & 73: Confirming that "Environmental change" is the intended phrase here, and not "global environmental change."	Environmental change is the intended phrase hence no need to change.
Line 144: I am wondering if all three of the reviewers initially reviewed separately and then came together to collaboratively come to a shared agreement? The iterative team approach could be described in more detail here.	We have further expounded on how the reviewers worked together in *Methods.* (two reviewers carried out the initial screening separately then the three reviewers worked together on the eligibility criteria and expansion of search using Google scholar).
Line 151: Search and screening strategy figure title could use more detail. For example, "...strategy for conducting a….". Also, can the "Screening of abstracts revealed limited geographic distribution" be connected to an additional or different step? At the moment it reads:<br><br>99 abstracts were screened, 77 were excluded, and from those that were excluded, their abstracts were screened again and revealed that there was a limitation in geographical distribution (which is surely not	We thank the reviewer for this observation and agree that a clarification on the figure 3 search and screening strategy is necessary in line with the correct explanation in Figure S2. Search strategy and search strings. The step on "Screening of abstracts revealed limited geographic distribution" was erroneously attached to the "excluded abstracts" which indeed is not the case. This step is now attached to the "Full articles included" which did reveal the limited geographic distribution.

the case here). Do the authors mean that they conducted an additional screening of the remaining abstracts to assess geographical distribution? Or that they conducted the initial screening of 99, and already found that the distribution was limited? Perhaps another arrow from the “abstracts screened” box directly to the “screening abstracts revealed…” box could clarify this in the figure?	
Line 158: Table 2- Row 1 Column 3 - “Geographic location of the Study” - is the capitalization of “study” intentional? If so, disregard the comment. If not, de-capitalize.	We have de-capitalised all the titles in the table for consistency.
Line 172: Table 2<br>What country did (29) get their funding from? At the moment the table states Asia.	The funding came from SEAMEO-BIOTROP which according to their website is based in Indonesia. We have therefore changed from Asia to Indonesia in the table.
(43) Could benefit from including China since not everyone would know what HSBC is. Assuming it is from China.	It is not clear where HSBC is based and no details are provided and we have sought not to make assumptions. As such we have not changed the text.
Figure 3: The Y-axis should be labeled.	As Figure 3 does not require a Y-axis, we assume the reviewer was referring to Figure 4 and have updated accordingly.
Line 358: Table 4 Caption could use more detail for it to stand alone. e.g. “a summary of the …..of citizen science data tools identified in a scoping review of….”	We have expanded the title for table 4 to “Categories of citizen science data tools from the reviewed studies”
Line 372: Can the authors indicate what “These” are? I suggest rephrasing to explicitly mention what they are referring to. E.g. “Proposals for data validation methods are evident…”	We have clarified the sentence editing to: “These proposals for data validation methods are evident in most of the reviewed studies to varying degrees.”
Ling 354: Section 3.7: This section could benefit from additional sub-headings such as Data collection + Data Validation and Verification. If three levels of headers are OK, please consider revising.	We agree that the previous sub-section on Data tools and Methods needs another subheading as it discusses both data tools and data validation. We have therefore split it into two subheadings – *3.7 Data tools and methods;* and *3.8 Data validation and verification.*
Line 381: The authors use et al on this line, can you consider using this naming in other parts of the manuscript where it can help with sentence flow? For example, Line 368 could flow better if it were Vattakaven et al.	We appreciate the reviewer’s expression of their style preference. But as this is a style preference, we will defer to the journal’s editorial team at the proof reading stage.

Line 443: Thank you for acknowledging the non-English exclusion limitation. Another sentence that explains what this exclusion may have missed could be useful here.	We have added the emphasis thus: "We acknowledge that this paper's exclusion of non-English articles perpetuates the hegemony of Anglo-American studies thus excluding other non-English knowledge systems.
Line 445: The authors state "that citizen science research in LMICs continues to be predominantly driven by institutions and funding agencies from the Global North…", but the abstract only talks about the predominance of funding. Is there a reason for leaving out the institutions part there?	The abstract synthesises the most significant aspects of the paper, thus emphasizing the predominance of Global North funding. This focus sets the stage to explore the connections between Global North institutions and funding agencies as we had expounded on in *Funding and lead institutions.*
Line 512: Table 5 caption can be more detailed<br>Is this an exhaustive list of environmental changes? As I look at the figure with "Environmental changes and ecosystem impairment," I am left to wonder if these were fully considered in the environmental changes list? Or if these indicators were just from the scoping review?<br><br>All of the categories could use the same level of definition that the authors provide for the "Direct health effects" category.	We have added some text for every illustrative category to improve comprehension.<br><br>The categories list is not an exhaustive list hence the use of the title "illustrative categories" to cater for updates to the exact categories as the literature evolves. But critically, the reporting indicators column of Table 5 is to improve the quality of reporting informed by the reporting inconsistency we found.
Grammar under the Human health outcomes list. eg should be e.g., - can this be applied throughout where it also applies?<br><br>For Demographics-Sex - could this exclude those who do not identify with a sex? Use of Examples may address this item (e.g., Male, Female, Other)	Eg changed to e.g. throughout<br><br>We have changed from Sex to *"Sex or Gender"* which will vary from study to study depending on the context and purpose of the study.
Discussion section considerations:<br><br>On the youth:<br>I note that several references involved youth and that the conclusion recommends " further research into how youth-driven citizen science initiatives can enhance and accelerate advancements in planetary health…" Given the conclusion's emphasis on youth, the findings and discussion could benefit from	We introduced the youth focus in the sub-section *Addressing planetary health through citizen science*. Investigation into youth involvement in the reviewed studies did not reveal meaningful results as only seven of the studies mentioned involving the youth in some capacity, with little elaboration on the significance of involving the youth. This informs our recommendations in the conclusion for further investigation into the significance of youth-driven citizen science in LMICs with the acknowledgment that LMICs have the most youthful urban populations globally.

additional detail that justifies the recommendation.	We have edited the conclusion to highlight this result gap and justify the recommendation with the following line added:<br>*"Lastly, a notable finding was a paucity of information on the (extent of) involvement of youth in studies identified. Given the rapidly growing youthful demographic in cities within LMICs,....."*
On regional unrest:<br>The authors note that regional unrest (Line 423, 502) was an external factor that posed a barrier in a study. Given that several LMICs experience some form of instability, particularly political instability, is there any relevance to including literature on this observation in the discussion? Upon searching "citizen science" in "fragile" contexts with limited government" on Google Scholar, I came across these references:<br><br>Fan, F. ti, & Chen, S. L. (2019). Citizen, Science, and Citizen Science. East Asian Science, Technology and Society: An International Journal, 13(2), 181–193. https://doi.org/10.1215/18752160-7542643<br><br>Benyei, P., Skarlatidou, A., Argyriou, D., Hall, R., Theilade, I., Turreira-García, N., Latreche, D., Albert, A., Berger, D., Cartró-Sabaté, M., Chang, J., Chiaravalloti, R., Cortesi, A., Danielsen, F., Haklay, M. (Mordechai) ., Jacobi, E., Nigussie, A., Reyes-García, V., Rodrigues, E., Sauini, T., Shadrin, V., Siqueira, A., Supriadi, M., Tillah, M., Tofighi-Niaki, A., Vronski, N. and Woods, T. (2023) 'Challenges, Strategies, and Impacts of Doing Citizen Science with Marginalised and Indigenous Communities: Reflections from Project Coordinators', Citizen Science: Theory and Practice, 8(1), p. 21. Available at: https://doi.org/10.5334/cstp.514.<br><br>*I am not asking to cite these above, rather see if there is any literature that can support this observation.	While we appreciate that several LMICs do experience political instability, only one of the reviewed studies mentioned regional unrest as a challenge for carrying out citizen science. As such, a discussion on instability is beyond the scope of this study.<br><br>Nonetheless, we have made reference to the need for further reporting on this with the following text under the "Researched Topics" section of the discussion:<br>"In addition, while only one reviewed study made reference to the context of regional unrest, the prevalence of political instability in many LMIC settings suggests the need for a better reporting and understanding of the practical experience of conducting citizen science research under such conditions."
